# System OMICs analysis of *Mycobacterium tuberculosis* Beijing B0/W148 cluster

**DOI:** 10.1038/s41598-019-55896-z

**Published:** 2019-12-17

**Authors:** Julia Bespyatykh, Egor Shitikov, Andrei Guliaev, Alexander Smolyakov, Ksenia Klimina, Vladimir Veselovsky, Maya Malakhova, Georgij Arapidi, Marine Dogonadze, Olga Manicheva, Dmitry Bespiatykh, Igor Mokrousov, Viacheslav Zhuravlev, Elena Ilina, Vadim Govorun

**Affiliations:** 1 Federal Research and Clinical Centre of Physical-Chemical Medicine, Moscow, Russian Federation; 2 Shemyakin-Ovchinnikov Institute of Bioorganic Chemistry of the Russian Academy of Sciences, Moscow, Russian Federation; 3 Moscow Institute of Physics and Technology (State University), Dolgoprudny, Russian Federation; 4 Research Institute of Phtisiopulmonology, St. Petersburg, Russian Federation; 5 St. Petersburg Pasteur Institute, St. Petersburg, Russian Federation

**Keywords:** Proteomics, Transcriptomics, Systems analysis

## Abstract

*Mycobacterium tuberculosis* Beijing B0/W148 is one of the most widely distributed clusters in the Russian Federation and in some countries of the former Soviet Union. Recent studies have improved our understanding of the reasons for the “success” of the cluster but this area remains incompletely studied. Here, we focused on the system omics analysis of the RUS_B0 strain belonging to the Beijing B0/W148 cluster. Completed genome sequence of RUS_B0 (CP020093.1) and a collection of WGS for 394 cluster strains were used to describe the main genetic features of the population. In turn, proteome and transcriptome studies allowed to confirm the genomic data and to identify a number of finds that have not previously been described. Our results demonstrated that expression of the *whiB6* which contains cluster-specific polymorphism (a151c) increased almost 40 times in RUS_B0. Additionally, the level of *ethA* transcripts in RUS_B0 was increased by more than 7 times compared to the H37Rv. Start sites for 10 genes were corrected based on the combination of proteomic and transcriptomic data. Additionally, based on the omics approach, we identified 5 new genes. In summary, our analysis allowed us to summarize the available results and also to obtain fundamentally new data.

## Introduction

*Mycobacterium tuberculosis* is one of the most dangerous pathogens. Every year more than 1.5 million people die from tuberculosis. Despite a decrease in new cases of active tuberculosis, the situation remains extremely tense due to the expansion of multidrug- and extensively drug-resistant strains, which represent a threat to the global tuberculosis control^1^. A large number of such resistant strains belong to the Beijing genotype, or more precisely, to the certain sublineages or clusters within the genotype. Of them, Beijing B0/W148 cluster attracts special attention. Initially, it was designated as B0^2^ and W148^3^ based on the IS6110 RFLP analysis. In recent classification it was called the Clade B^4^, ECDC0002^5^, East European cluster 2^6^, or CC2^7^.

Currently, members of this cluster comprise one-fourth of all Beijing genotype isolates in Russia and in some former Soviet Union countries^8^. Also Beijing B0/W148 isolates recovered from Russian immigrants^9,10^. In addition to high drug resistance, these isolates exhibit extra features in comparison to representatives of other genotypes, such as increased virulence and transmissibility, and overall fitness success.

Members of the Beijing B0/W148 cluster are most fully studied at the genomic level. In particular, the phylogenetic position of this group within the lineage 2 and the main cluster-specific polymorphic sites have been determined^7,11^. Additionally, all strains possess large chromosomal rearrangements in the genome^12^. This is an unusual event for the *M. tuberculosis*, since recombination events are extremely rare in this pathogen, and previously only cases of duplication of extended regions have been described^13^. Notably, Beijing B0/W148 strains carry a specific frameshift mutation in the *kdpD* gene (c. 2541_2542delCA), which leads to the formation of the fusion gene *kdpDE*. Both *kdpD* and *kdpE* belong to the two-component system and a partial deletion of the *kdpDE* operon in *M. tuberculosis* has already been associated with enhanced virulence^14^. As a result of such in-depth genetic analysis, a number of methods for identification of the cluster strains based on PCR^15^ and biochip^16^ have been developed.

To date, results of only a few proteomic studies for members of the cluster have been reported. De Keijzer *et al*. determined the temporal dynamics of the proteome and corresponding Ser/Thr/Tyr phosphorylation patterns of *M. tuberculosis* Beijing strain B0/W148 in response to rifampicin^17^. In our laboratory we carried out a total proteomic analysis of the cluster strains under physiological conditions^18^ and demonstrated low abundance of the DosR regulon proteins compared to the H37Rv strain in the stationary phase. At the same time, an increase representation of proteins of this regulon was detected under stress conditions and antibiotic treatment^17^. Earlier studies reported conflicting data of mRNA level for the *dosR* regulon genes in this specific genotype^19,20^. However, it should be noted, that all the discussed studies were performed on different independent cluster strains. Currently, the NCBI database contains only one complete genome of the strain W-148 (GenBank, CP012090.1), belonging to the Beijing B0/W148 cluster. This strain poorly characterized and the systemic analysis of the cluster is problematic.

In this study we conducted a comprehensive investigation of the *Mycobacterium tuberculosis* Beijing B0/W148 cluster based on the multi-omics analysis of its member - RUS_B0 strain. Whole genome sequencing, total proteomic and transcriptomic analyses were performed, which allowed us to make the most complete description of the cluster to date.

## Results

### Phenotyping and genotyping features

**Figure 1:**
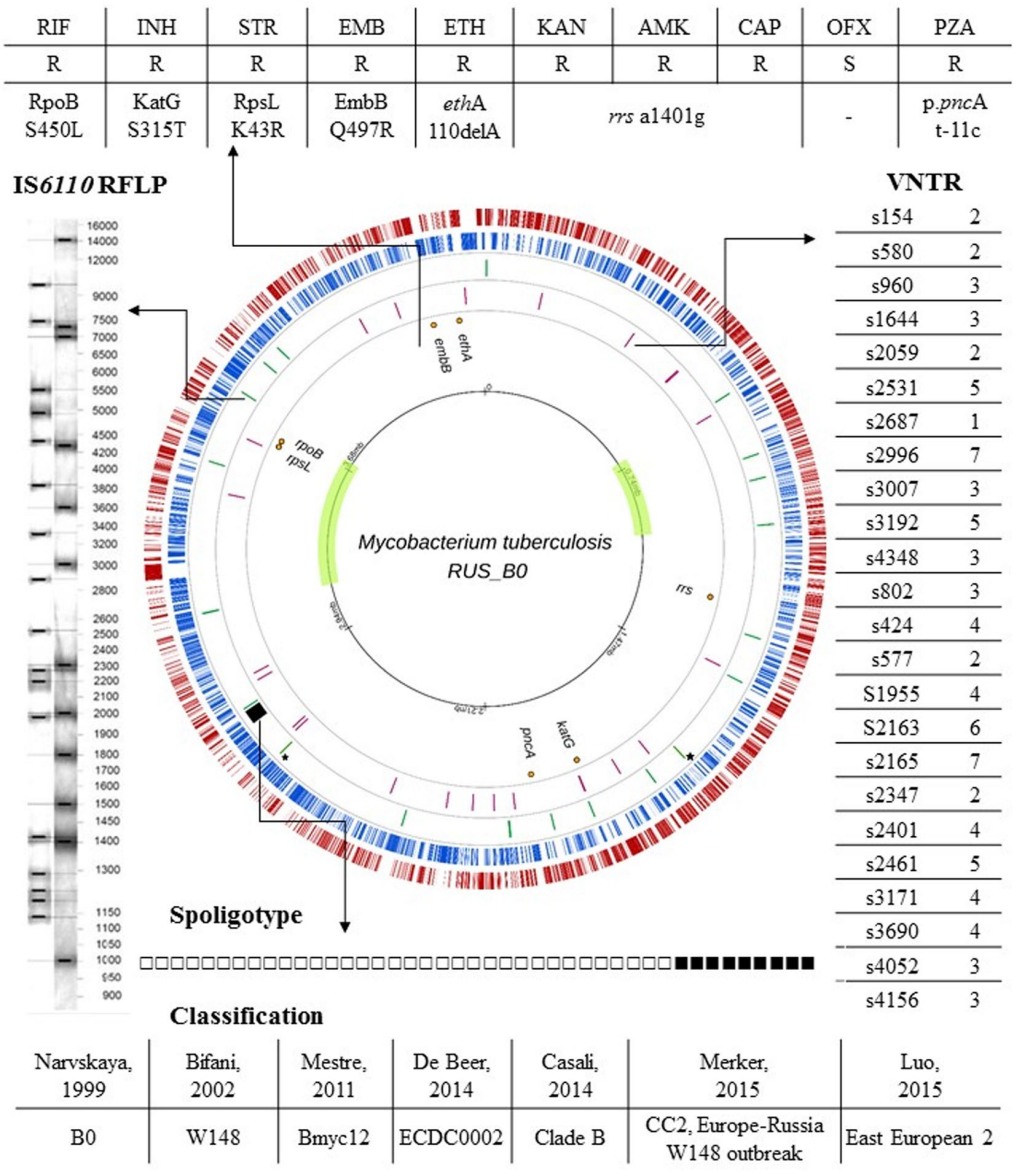
Circular map and genetic features of *M. tuberculosis* RUS_B0. Two outer circles show the coding sequences on plus (red) and minus (blue) strands; the third ring depicts CRISPR locus (black) and IS6110 integration sites (green) (Beijing B0/W148 specific sites indicated by asterisk); the fourth ring shows the positions of the VNTR; the fifth ring shows drug resistance genes; and the sixth ring shows the scale in the genome with the inverted regions with respect to the H37Rv genome.

The *Mycobacterium tuberculosis* RUS_B0 strain was isolated in the Research Institute of Phthisiopulmonology (St. Petersburg, Russian Federation) in 2008 from a patient with infiltrative tuberculosis of the upper lobe of the left lung. Detailed strain characteristics are presented in the Fig. 1. Spoligotyping analysis revealed that RUS_B0 strain belonged to the Beijing spoligotype (SIT1). The 24 loci MIRU-VNTR and IS6110 RFLP typing methods showed the patterns specific for Beijing B0/W148 cluster. The latter was also confirmed by PCR. According to the drug susceptibility testing, the strain belonged to pre-XDR tuberculosis and was resistant to streptomycin, isoniazid, rifampicin, ethambutol, ethionamide, pyrazinamide, kanamycin, amikacin, and capreomycin. Growth kinetics of the RUS_B0 and H37Rv strains in liquid cultures is presented in Supplementary Fig. S1.

### Genomic characteristics of the cluster

The resulting (circular) genome of RUS_B0 (CP030093.1) was 4,418,559 bp in length with an average GC content of 65.6%. One set of rRNAs and 45 tRNA genes were identified in the genome, as well as 4,115 protein-coding genes and 138 pseudogenes.

At least 1,395 isolated SNPs compared to the H37Rv genome were revealed: 458 and 732 of them were identified as synonymous and non-synonymous SNPs, respectively. We found all SNPs characteristic for lineage 2, as well as for modern Beijing and Beijing B0/W148 cluster (Table S1).

**Figure 2:**
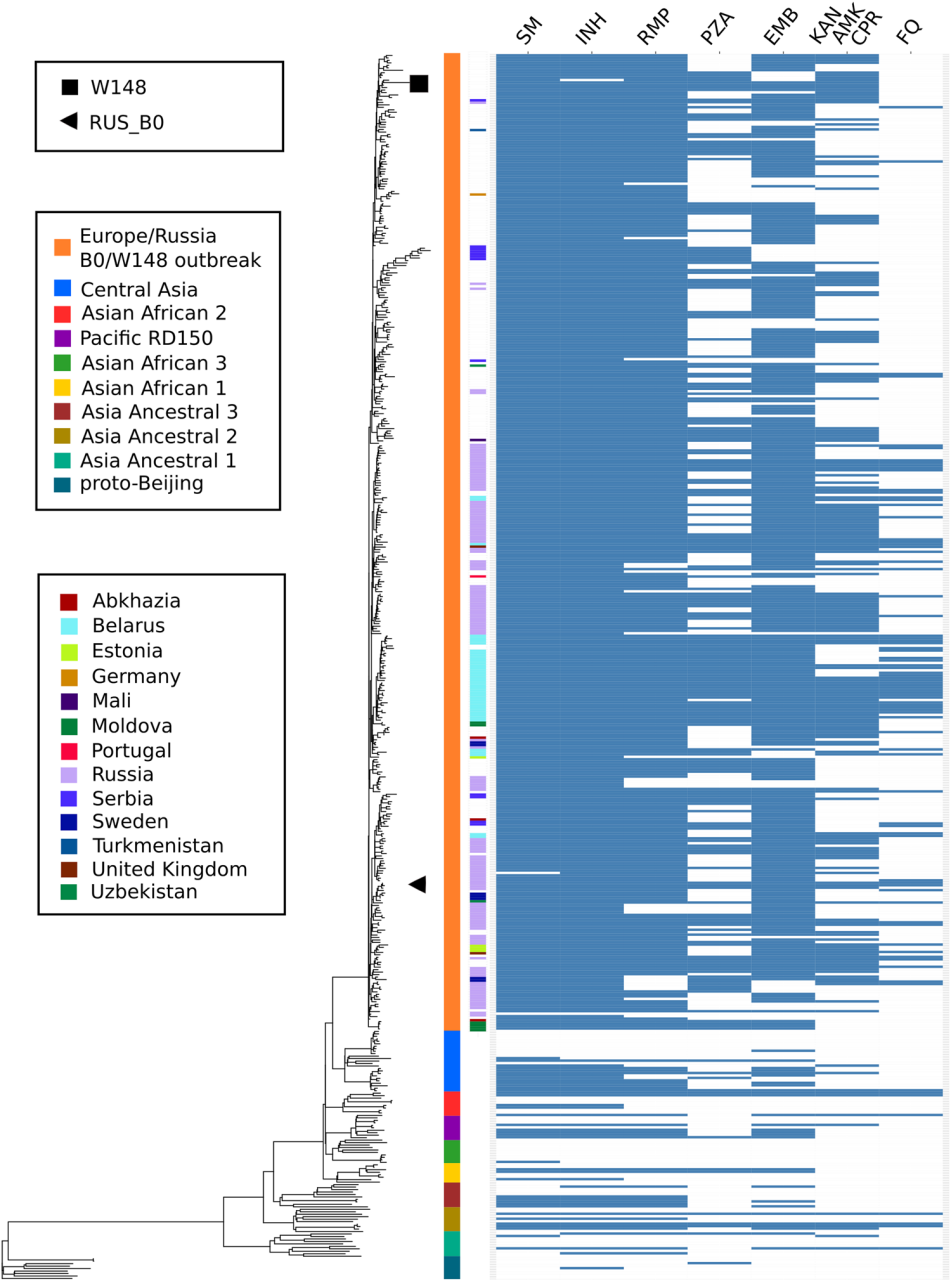
Phylogeny of Beijing B0/W148 isolates. A total of 496 genomes were used to reconstruct a maximum-likelihood phylogenetic tree. Columns show genetic drug resistance to first- and second-line drugs^40^. Samples from different sublineages of lineage 2 were used to define phylogenetic relationships. SM = streptomycin, INH = isoniazid, RMP = rifampicin, PZA = pyrazinamide, EMB = ethambutol, KAN-AMK-CPR = kanamycin-amikacin-capreomycin, FQ = fluoroquinolone.

To infer position of RUS_B0 strain on the phylogenetic tree and its relationship with other members of the Beijing B0/W148 cluster, we performed a phylogenetic analysis. For this, we randomly selected 10 samples for each described sublineage of lineage 2^21^, as well as the maximum number of Beijing B0/W148 cluster strains from NCBI. Overall, 496 strains were used to construct a SNPs-based phylogenetic tree (Fig. 2, Table S2).

On average, the cluster strains differed from the reference genome H37Rv by 1,600 SNPs (range from 1,165 to 1,870) and mean pairwise distance between the cluster strains was 47.21 SNPs ± SD 13.16. The latter corresponds to the hypothesis of their recent formation^7^.

We noted a high level of drug resistance (5.74 ± SD 1.99) of the cluster samples, as well as a good correlation of the origin of patients with some branches of the phylogenetic tree. Cluster strains were identified in 13 countries. RUS_B0 strain was in one group with samples from Uzbekistan (ERR552580), Germany (ERR551987, ERR551986, ERR552451, ERR551063, ERR551062; place of patient birth not determined), and Sweden (SRR1162700, SRR1166927, SRR1166707, place of patient birth not determined). In turn, the W-148 (CP0012090.1) strain differed from the RUS_B0 (CP030093.1) by 43 SNPs and clustered within another group on the phylogenetic tree (Fig. 2; Table S3).

### Transcriptomic analysis

**Figure 3:**
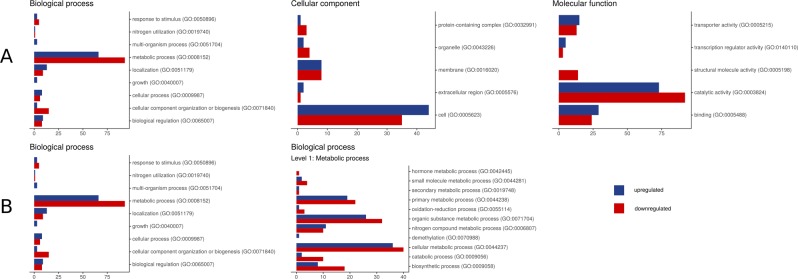
Multi-omics analysis of *ethA* gene and its product in H37Rv and RUS_B0 strains. The horizontal axis represents a schematic *ethA* gene (cyan) and protein (yellow), vertical axis represents transcript coverage (blue) and peptides (green). The red square indicates a frame shift mutation in the RUS-B0 strain genome.

Transcriptomic analysis resulted in the identification and quantification of 3,887 and 3,906 transcripts (TPM > 1) for the RUS_B0 and H37Rv, respectively (Table S4). Of them, 830 genes had statistically significant differences in expression between the RUS_B0 and H37Rv strains (FDR ≤ 0.01, fold change ≥2) (Table S5). As expected, the most significant changes in expression were noted for the genes belonged to the regions of differences (RD and RvD for RUS_B0 and H37Rv, respectively). These regions are absent in the strains and, accordingly, gene expression does not occur. Among the RUS_B0 up-regulated genes, the high level of expression of *whiB6* (40-fold) and *ethA* (8-fold) should be mentioned. Transcriptional regulator WhiB6 is a part of PhoP regulon and has a cluster-specific substitution A253S. EthA encodes a monooxygenase, which is required for activation of the prodrug, ethionamide. RUS_B0 strain carries a frame shift mutation in the *ethA* gene (c. 110delA) that leads to the subsequent formation of stop codons (Fig. 3).

**Figure 4:**
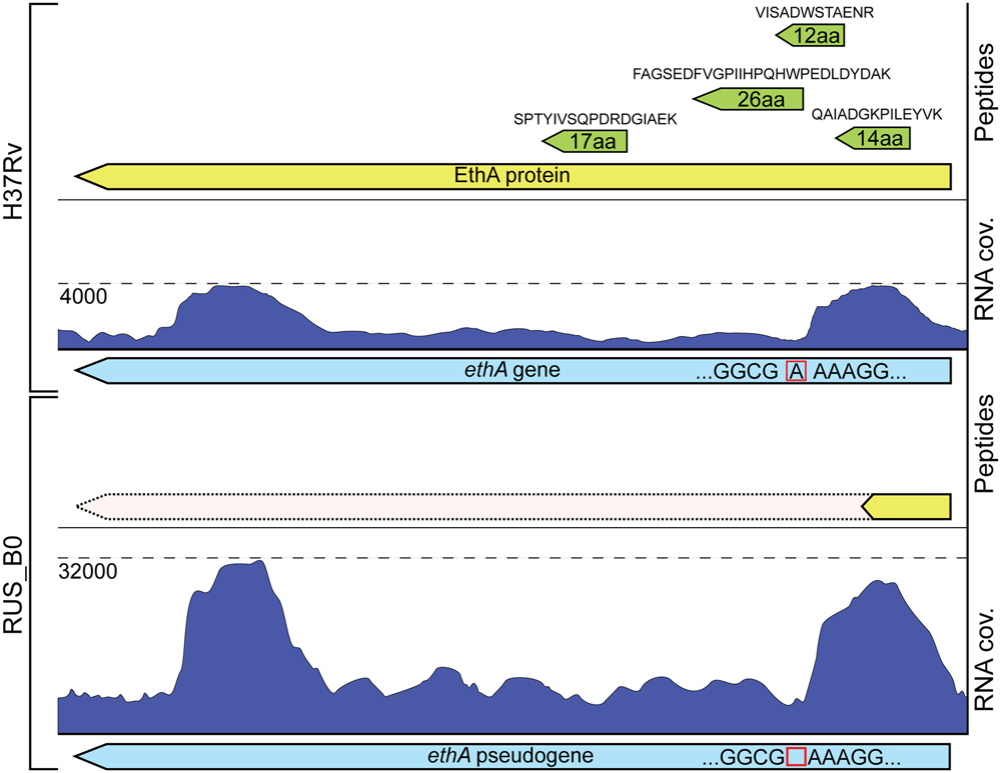
Representative functional clusters for differential genes based on transcriptomic data. Blue color indicates down-regulated genes. Red color indicates up-regulated genes. (**A**) Distribution of genes with diff expression in the main categories; (**B**) Detail representation of genes with diff expression in «biological process» category.

The GO-analysis revealed that in the category of Molecular Functions up-regulated genes were more often found in groups Transporter activity (GO:0005215) and Binding (GO:0005488). While down-regulated genes prevailed in groups Catalytic activity (GO:0003824) and Structural Molecule Activity (GO:0005198) (Fig. 4А). In the category of Biological Process, most of the down-regulated as well as up-regulated genes belonged to the group Metabolic Process (GO:0008152). It is interesting to note that in most groups down-regulated genes prevailed, while more up-regulated genes were found in the Nitrogen Compound Metabolic Process (GO:0006807) group. Additionally, category Demethylation (GO:0070988) contained only up-regulated genes (Fig. 4B).

### Proteomic and proteogenomic analysis

A total of 158,485 MS/MS spectra were acquired during proteomic analysis, of which 14,056 were assigned to unique peptide sequences (FDR < 1%). For protein identification, both RUS_B0 and H37Rv proteomic databases were used.

**Figure 5:**
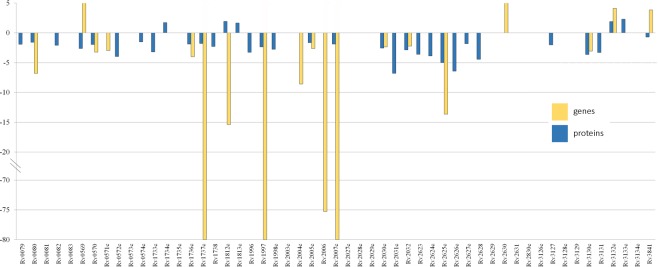
DosR regulon expression and protein level. Bars indicate fold changes in gene expression (blue bars) and protein level (yellow bars) in RUS_B0 relative to the same genes in the H37Rv strain (x axis). Stars indicate the proteins which were found in only one strain.

In total, 1,977 proteins (about 50% of the total cell proteome) were identified. For all detected proteins functional categories (TubercuList) and subcellular localizations (PSORTdb) were established (Table S6). For quantitative proteomic analysis the H37Rv proteome data (PXD002542) were used^18^. In summary, 511 and 875 proteins were over- and underrepresented in the RUS_B0 strain, respectively (Table S7). The main changes detected in DosR regulon system proteins (Rv0080, Rv0570, Rv0571c, Rv1735c, Rv1737c, Rv1812c, Rv1997, Rv2004c-Rv2007c Rv2030c, Rv2032, Rv2625c, and Rv3130c) which had low abundance in the cluster strain (Fig. 5). The high representation of cellular lipid metabolic process (GO:0044255) (Rv0632c, Rv0971c, Rv1142c, Rv1472, Rv3039c, Rv3373, Rv3516) in RUS_B0 has been confirmed as compared to H37Rv^18^.

For further proteogenomic analysis we conducted six-frame translation directly of RUS_B0 genome. As a result, 38 GSSPs corresponding to 24 ORFs were identified. Of those, 13 ORFs (14 GSSPs) contained already annotated genes and 11 ORFs (24 GSSPs) corresponded to new, previously not annotated genes. After three-frame RNA-Seq transcript translation 10/14 and 10/24 GSSPs were confirmed (Table 1). For full results of blast similarity search see Supplementary Table S8.

**Table 1:** GSSPs validated in *M. tuberculosis* RUS_B0 strain.

RUS_B0 locus	Locus in H37Rv	Six prot acc	Unique GSSP	Previous aminoacid	GSSP sequence	Next aminoacid	Best Mascot Ion score	Best Mascot Identity score	Best Mascot Delta Ion score	Best X! Tandem –log (e) score	Annotated in other *M. tubeculosis* strains	Found in transcriptome data
**Start site correction**												
DPM14_06085	Rv1330c	1279837:1281369_reverse	1	R	TAVGPPPAAR	R	50,2	25	36,7	1,19	Yes	No
DPM14_06590	Rv1425	1379719:1381659_forward	1	R	IAVSDPHQVLQAVIR	P	0	0	0	1,6	No	No
DPM14_07380	Rv1567c	1557336:1557938_reverse	1	R	SALDEPDER	T	37,5	25	28,6	0	Yes	Yes
DPM14_01015	Rv0186	214254:216599_forward	1	R	EVEAQMTDDER	F	22,8	25	22,8	1,41	Yes	Yes
DPM14_15480	Rv1093	3192084:3193553_reverse	1	R	TTAVMSAPLAEVDPDIAELLAK	E	32,3	25	32,3	7,7	Yes	Yes
DPM14_16025	Rv0998	3298349:3299428_reverse	1	R	PDATTVRTTVSWAWR	R	28,8	25	28,8	0	Yes	No
DPM14_18255	Rv3329	3713427:3714839_forward	1	R	SLPEPSAALANTTTR	N	15,5	25	15,5	2,01	Yes	Yes
DPM14_18270	NA	3716955:3718877_forward	1	R	AAVHHRVQLR	I	17	25	14	0	No	Yes
DPM14_19510	Rv3556c	4002140:4003402_reverse	1	R	GAVMTEAYVIDAVR	T	0	0	0	1,28	Yes	Yes
DPM14_19610	Rv3574	4021857:4023419_forward	1	K	VAVLAESELGSEAQR	E	46,2	25	46,2	2,6	Yes	Yes
DPM14_19975	Rv3646c	4091010:4094006_reverse	1	R	PLPTGAVSIGAGIR	D	0	0	0	1,08	Yes	Yes
DPM14_20195	Rv3684	4132188:4133507_forward	2	R	LIEADAR	R	22,3	26,8	10,4	1,07	Yes	Yes
				R	SWTDNAIR	L	24,9	25	9,15	0	Yes	Yes
DPM14_21155	Rv3856c	4335338:4336528_reverse	1	R	ATGVSPIMDPVTALR	Q	14,4	25	13,1	1,11	Yes	Yes
**New genes**												
NA	NA	104947:105810_reverse	2	R	LVLRIELSGR	G	0	0	0	1,59	No	Yes
				R	NDLSNYPAEFDELPR	R	0	0	0	1,18	Yes	Yes
NA	NA	1075075:1075662_reverse	2	R	EPVGVLGSGIGR	N	20,3	25	15,4	0	Yes	Yes
				R	RGGGGVLR	G	16,7	25	−1,05	0	Yes	Yes
NA	NA	1095047:1095517_reverse	2	R	IPQRCGVIR	N	27,9	25	11,9	1,44	Yes	No
				R	IHQAPIRVLDR	E	21,8	25	20,4	0	Yes	No
NA	NA	1680143:1680568_reverse	2	-	NAVSSPGAGLATVAATR	T	0	0	0	1,59	Yes	No
				R	PLNTMPLVR	T	15,9	25	0	0	Yes	No
NA	NA	1733676:1734110_reverse	2	R	PIPPIGALR	L	24,9	25	0	0	Yes	No
				R	SGAASSGAR	P	21,8	25	5,72	1,16	Yes	No
NA	NA	1958092:1958697_forward	2	R	VAGPSNPELPGHPHPCR	G	0	0	0	1,24	Yes	No
				R	DRPAGQPVR	R	26,9	25,3	5,29	0	Yes	No
NA	NA	2830724:2831131_forward	2	R	VHNLDPELVDEHAR	Q	34,9	25	33,3	5,77	Yes	No
				R	YGPRPDPND	-	0	0	0	1,57	Yes	No
NA	NA	2911505:2912077_reverse	2	R	RSLLAMASPR	R	37,4	25	10,8	0	No	No
				R	SLLAMASPR	R	37,4	25	10,8	0	No	No
NA	NA	3117184:3117807_reverse	2	R	IPSEVDGLLR	L	22,7	25	17,2	0	Yes	Yes
				R	HAAQRVVGVAVFK	Q	14,8	25	13,3	0	Yes	Yes
NA	NA	3766655:3767689_forward	2	R	AGGQAQVGKPQVAMADVAQPQVAVPDIAQAQVVPTDVGR	T	0	0	0	1,24	Yes	No
				R	VEATGVGVAGVEAARVVVTSVGDAGAEAAGIEEPGAGR	S	0	0	0	2,28	Yes	No
NA	NA	3999293:4001251_forward	4	R	GAGSIAGR	V	30	25	14,8	0	Yes	Yes
				R	HPAQLLDLR	P	26,5	25	21,5	0	Yes	Yes
				R	KPIHADLR	Q	28,3	25	21,5	0	Yes	Yes
				R	HLHAVRAAGDIR	S	14,8	25	14,8	0	Yes	Yes

## Discussion

Beijing B0/W148 cluster is perhaps the most mentioned population in the epidemiological surveys conducted in Russia, Eastern Europe and Central Asia. It was “re-discovered” and “re-named” at least 7 times. Its distribution, origin, phenotypic traits and drug resistance were discussed in a recent review^8^. In turn, this study was focused on genomic, transcriptomic and proteomic features of the cluster strains.

For this purpose, we selected RUS_B0 strain as a typical member of the cluster (Fig. 1). The strain was resistant to 9 drugs and, according to this indicator, was slightly different from the average for the cluster. It is worth noting that we identified only one strain that was sensitive to all antibiotics (SRR6353889, extrapulmonary tuberculosis, collection data: 2011, place of collection: St. Petersburg). This implies that this patient was infected before the spread of the resistant clone. Interestingly, we also found that several cluster strains carry a rifampicin resistance mutation other than S450L (36 of 369; 9.8%) (Fig. S2). During the study, we also analyzed compensatory mutations that occur after a mutation in the RRDR region of the *rpoB* gene^22,23^. In our dataset, about 70% (260 of 369) of the RIF resistant strains carried hypothetical compensatory mutations in *rpoA*, *rpoB*, and *rpoC* genes (Table S9). It should be noted that for countries with a high burden of the disease, this indicator usually does not exceed 40%^22^. The strain under study also carried a compensatory mutation in the *rpoC* gene (position; G332S), which could affect its fitness. However, the RUS_B0 strain had a slightly lower growth rate compared to the H37Rv (Fig. S1). Most likely this is due to the fact that RUS_B0 was also resistant to other drugs and its fitness was still lower than that of the laboratory strain. It should also be noted that we did not reveal differences at the level of expression and abundance of RNA polymerase between the two strains.

Analyzing resistance of the RUS_B0 strain to ethionamide, a frame shift mutation in the *ethA* gene was identified (ct4327363c; c. 110delA). Earlier it was shown that a mutation in its promoter region results in the functionally inactive enzyme and subsequent drug resistance^24^. Similarly, low level of *ethA* transcription was reported in the isoniazid-resistant strains^25,26^. According to our data, the level of *ethA* transcripts in RUS_B0 was increased almost 8 times compared to the H37Rv. However, we didn’t identify corresponding protein EthA (GO:0003824) in RUS_B0. After a frame shift mutation in *ethA* gene a lot of stop codons were formed. Therefore, gene was not functional and corresponding protein not synthesized (Fig. 3).

At the same time, an increased abundance of another monooxygenase, MymA (Rv3083), was detected, which, in our opinion, can replace the functions of the EthA in the bacterial cell. Should be noted, genes of the operon mymA (Rv3083-Rv3089) and its regulator *vir*S (Rv3082c) are activated in response to changes in pH (for example, in the host under the influence of adverse environmental conditions)^27^. Presumably, MymA (GO:0003824) might play a role in the modification of fatty acids needed for the enveloping of *M. tuberculosis*. In our study, under normal conditions, low expression of the *virS* and *mymA* was documented in the RUS_B0 strain. This may be due to the fact that the cluster strains have low fitness; however, they are able to quickly respond to the changing environmental cues. In turn, the latter may cause increased virulence, which is one of the main characteristic of a cluster^8^.

Significant differences were also found in the expression level of the DosR regulon genes. This regulon includes 53 genes, the expression of 20 of which in the RUS_B0 was two-fold or lower compared to the H37Rv (Table S5). The expression of another 13 genes was also lower in the cluster strains. Number of DosR regulon proteins were also under represented in RUS_B0 (Fig. 4) which again agrees with the previously obtained proteomic data^18^. At the same time, a number of transcriptome^13,19,28^ and proteome^29^ studies reported an increased representation of the DosR regulon genes and proteins in cluster strains compared to H37Rv. Earlier we suggested that Beijing strains implement an alternative response to hypoxic stress than that used by H37Rv according to DosR proteins level^18^. Data obtained in this study may also indicate differences in the metabolic pathways associated with oxygen. Notably, the level of the transcript of the regulator itself (TF), *dosR* gene, was higher in the RUS_B0 compared to the H37Rv. Presumably, despite the high representation of the transcript, phosphorylation of the protein DosR does not occur and, accordingly, there is no activation of transcription of the regulon genes directly.

Importantly, the expression of the *whi*B6 (Rv3862c) was increased almost 40 times in the RUS_B0. This gene contains a cluster-specific polymorphic site, leading to the amino acid substitution A253S in the appropriate protein. We hypothesize that this mutation leads to a decrease in the functional activity of the enzyme, causing a compensatory increase in the level of the corresponding gene expression. Presumably, *whi*B6 involved in the regulation of redox balance in the cell^30^. Such regulation is carried out with the participation of DosR regulon, which enables to establish a persistent infection and maintain the integrity of granulomas.

Finally, multi omics data allowed to confirm our previous findings. Earlier in the Beijing B0/W148 cluster strains we detected high and low abundance of fatty acid synthesis and degradation enzymes, corresponding^18^. Additionally, in the RUS_B0 strain compared to H37Rv a reduced expression of a large number of genes responsible for the synthesis of proteins involved in the degradation of fatty acids was detected. At the same time transcripts and proteins corresponding to Transporter activity (GO:0005215) were over represented in RUS_B0 which is consistent with previously published data^18^. We also observed a reduced expression of the *sseA* (Rv3283), which showed a low representation at the proteomic level^18^. The latter completely correlates with the results of other Beijing B0/W148 cluster strains studies^31^.

Carrying out of the whole genome sequencing of the RUS_B0 strain allowed us to correct a number of inaccuracies present in the annotation of the W-148, the only previously sequenced member of the Beijing B0/W48 cluster^32^. For example, the peptides AASSLSGGADTLEALGVR and IDLGPDLVAR, previously identified as parts of the of the pseudogene TBPG_RS21255 in W-148 by using H37Rv annotation, do belong to the TrpD anthranilate phosphoribosyltransferase (DPM14_10670) in RUS_B0. Withal, LTSAGDDAERSDEEER and RLTSAGDDAER peptides have been previously identified only after six-frame translation as GSSPs. In the RUS_B0 annotation, the corresponding peptides were identified for the DUF3000 domain-containing protein (DPM14_13425). It has been proposed earlier that the TBPG_RS19380/Rv3684 might have an alternative start^32^. In the present study, we confirmed this conclusion, as we identified GSSP SWTDNAIR and new GSSP LIEADAR peptides in our proteomic dataset. According to three-frame RNA-Seq translation, transcripts for these GSSPs were also found (Table 1). For all the putative new genes documented in this study, at least two GSSPs were identified. For most of these genes, homologs in other strains of *M. tuberculosis* were easily detectable in the NCBI database. However, only four of them were confirmed by transcriptomic data (Table 1).

## Conclusions

In this study, for the first time, the most comprehensive description of the *M. tuberculosis* Beijing B0/W148 cluster was presented. Combined analysis of RUS_B0 genome and a large collection of the WGS allowed us to characterize the main markers of the cluster. At the same time, proteomic and transcriptomic data demonstrated not only their usefulness for validation and correction of genomic information, but also acted as independent sources of valuable information. Our analysis allowed us to summarize the available and also to obtain fundamentally new data. Undoubtedly, such a work on collecting multi-omics data in one strain open numerous opportunities for more comprehensive analysis of this diseases.

## Materials and Methods

### *Mycobacterium tuberculosis* strain and growth conditions

The RUS_B0 (earlier name 1947) (Beijing B0/W148 cluster) and the reference H37Rv strains of *Mycobacterium tuberculosis* were used. For proteomic and transcriptomic studies, *M. tuberculosis* strains were grown without shaking in liquid Middlebrook 7H9 medium with OADC supplement (Becton Dickinson, USA) at 37 °C in three biological replicates. To determine the growth rate, strains in 9 biological replicates were cultured in Mycobacterial growth indicator tubes (MGIT) on a BACTEC MGIT automated Mycobacterial detection system (Becton Dickinson) at 37 °C for 6–7 days. The susceptibility testing was done using the BACTEC MGIT 960 Culture system (Becton Dickinson) following the manufacturer’s protocol. Manipulation of cultures were performed in a BSC II (Thermo Fischer Scientific Inc., USA) within a TB-containment laboratory.

Whole-genome sequencing (WGS) data of 5,715 *M. tuberculosis* isolates was obtained from the National Center for Biotechnology Information (NCBI) and European Nucleotide Archive and used for B0 samples identification as reported previously^11^.

### Genomic analysis

#### Genotyping

Genomic DNA was isolated from *M. tuberculosis* RUS_B0 strain using standard extraction method^33^.

To verify that the strain belongs to a Beijing B0/W148 cluster, the PCR assay was performed as in^15^. Spoligotyping, IS6110-RFLP and 24-MIRU-VNTR typing were performed as described in^16,31^ and^32^, respectively.

#### Whole-genome sequencing and analysis

DNA was used for library preparation and sequenced using Illumina HiSeq. 2500 Sequencing Platform following the manufacturer’s instructions. Raw data were deposited in the NCBI Sequence Read Archive under accession number PRJNA421323. WGS reads were aligned to the *M. tuberculosis* H37Rv (NC_000962.3) and W-148 (CP012090.1) genome sequences using Bowtie 2^34^. SAMtools (v.0.1.18) and FreeBayes (v.1.1.0) were used for variant calling^35,36^. For FreeBayes, SNPs with a minimum mapping quality of 20, minimum coverage of 10 and alternate fraction of 0.9 were taken.

To assemble the genome, SPAdes v3.5.0 was used with read error correction and mismatch careful modes on^37^. Additionally, K-mer values were selected automatically based on maximum read length. To assess the quality of the assembly, QUAST v4.6.1 was used^38^ with W-148 genome as a reference. To determine the full-length genomic sequence of RUS_B0, the regions between contigs corresponding to repetitive regions or regions with no coverage were additionally sequenced on ABI Prism® 3730 Genetic Analyzer (Applied Biosystems, USA; Hitachi, Japan). The resulting (circular) genome of RUS_B0 was deposited in the NCBI under accession number CP030093.1.

Phylogenetic tree was built based on overall SNPs after excluding repetitive, mobile elements, PE-PPE-PE_RGRS, drug-resistance associated genes and artifact SNPs linked to indels using RAxML v8.2.11^39^ under GTR-CAT model with ascertainment bias correction. A comprehensive list of drug-resistance mutations to first- and second-line drugs was used to determine genetically resistant strains^40^. The identification of IS6110 integration sites was carried out using ISMapper pipeline^41^.

### Transcriptomic analysis

#### RNA extraction

Bacterial pellets were resuspended in Trizol LS and 0.5 mm silica-zirconium beads. Cells were disrupted using a bead-beating homogenizer (MPBio, FastPrep-24, USA) for 4 min, followed by 5 min incubation on ice. Nucleic acids were extracted with phenol-chloroform-isoamyl alcohol (25:24:1) and RNA was precipitated with 0.4 volume of absolute ethanol. Total RNA was purified three times with Trizol reagent (Invitrogen, USA), RNA integrity was analyzed with a Bioanalyzer (Agilent Technologies, USA), quantified by spectrophotometry with the NanoDrop ND-1000 (Thermo Fisher Scientific, USA).

DNase treatment carried out with TURBO DNA-free kit (Thermo Fisher Scientific) in volume 100 µL and then On-Column DNase Digestion with the RNase-Free DNase Set (Qiagen, Germany) according to manufacturer’s protocol. RNA cleanup was performed with the RNeasy Mini Kit (Qiagen) according to the RNA Cleanup protocol. DNA contamination was evaluated by PCR, using primers for amplification of the IS6110 fragment (PolyTub, Lytech, Russia). Finally, RNA was dissolved in DEPC-treated water and stored at −70 °C until further use. Concentration and quality of the total extracted RNA was checked by using the Quant-it RiboGreen RNA assay (Thermo Fisher Scientific) and the RNA 6000 pico chip (Agilent Technologies), respectively.

#### RNA-seq and analysis

Total RNA (1–2.5 µg) was used for library preparation. Ribosomal RNA was removed from the total RNA and libraries were prepared using the ScriptSeq Complete kit (Epicentre, USA), according to manufacturer’s protocol. Subsequently, RNA cleanup was performed with the Agencourt RNA Clean XP kit (Beckman Coulter, USA). The library underwent a final cleanup using the Agencourt AMPure XP system (Beckman Coulter) after which the libraries’ size distribution and quality was assessed using a high sensitivity DNA chip (Agilent Technologies). Libraries were subsequently quantified by Quant-iT DNA Assay Kit, High Sensitivity (Thermo Fisher Scientific). Finally, equimolar quantities of all libraries (12 pM) were sequenced by a high throughput run on the Illumina HiSeq using 2 × 125 bp paired-end reads and a 5% Phix spike-in control. Before loading the cBot system, the libraries were incubated at 98 оC for 2 minutes and then cooled on ice to improve hybridization of the GC-rich sequences. In total, 114 and 110 million paired reads were obtained corresponding to 14 and 13 billion nucleotide bases for H37Rv and RUS_B0 respectively. Dataset of RNA-Seq analysis have been deposited to the NCBI with project name PRJNA421323.

To map RNA-Seq reads, Kallisto software was used^42^. Differential expression analysis was performed using edgeR implemented in Degust web-tool. Significance cut-off was set at FDR < 0.01 and minimum expression fold change more or equal to 2. The number of reads mapping to each coding sequence (CDS) was calculated and normalized for gene length and library depth to generate transcripts per million (TPM).

### Proteomic analysis

#### Protein extraction and trypsin digestion

For proteomic analysis, the bacterial cells were harvested by centrifugation at 3,500 rpm for 5 min and washed with Tris-HCl and 2% Triton-X100 (pH 7.5–8). Cells were precipitated by centrifugation at 4,500 rpm, 4°С for 15 min and stored at −80 °C until required. The bacterial pellet was lysed and protein extracted as described previously^18^. Protein concentration was measured by the Bradford method using the Bradford Protein Assay Kit (Bio Rad, USA)^43^.

Proteolytic in-gel digestion was performed in three biological and two technical replicates: (1) sample, fractionated into 6 parts; (2) total load sample as described previously^32^. Peptides were cleaned using C18 Sep-Pak columns (Waters, USA)^18^.

#### LC-MS/MS analysis

Peptides were separated with high-performance liquid chromatography (HPLC, Ultimate 3000 Nano LC System, Thermo Fisher Scientific,) in a 15-cm long C18 column with a diameter of 75 μm (Acclaim® PepMap™ RSLC, Thermo Fisher Scientific). The peptides were eluted with a gradient from 5 to 35% of buffer B (80% acetonitrile, 0.1% formic acid) over 115 min at a flow rate of 0.3 μl/min. Total run time including 15 min to reach 99% buffer B, flushing 10 min with 99% buffer B and 15 min re-equilibration to buffer A (0.1% formic acid) amounted to 65 min. Further analysis was performed with a Q Exactive HF mass spectrometer (Q ExactiveTM HF Hybrid Quadrupole-OrbitrapTM Mass spectrometer, Thermo Fisher Scientific). Mass spectra were acquired at a resolution of 60,000 (MS) and 15,000 (MS/MS) in a range of 400–1,500 m/z (MS) and 200–2,000 m/z (MS/MS). An isolation threshold of 67,000 was determined for precursors selection and up to top 25 precursors were chosen for fragmentation with high-energy collisional dissociation (HCD) at 25 V and 100 ms activation time. Precursors with a charged state of + 1 were rejected and all measured precursors were excluded from measurement for 20 s. From 2 to 4 technical runs were analyzed for each sample.

#### Protein identification and quantitation

Raw data was captured from the mass spectrometer and converted to MGF (Mascot Generic Format) files using ProteoWizard with the following parameters: peakPicking true 2, msLevel 2, zeroSamples removeExtra^44^. For thorough protein identification, the generated peak lists were searched with the MASCOT (version 2.5.1, Matrix Science Ltd, UK) and X! Tandem (VENGEANCE, 2015.12.15, The Global Proteome Machine Organization) search engines. Database-searching parameters were as following: tryptic hydrolysis, no more than one missed site, the precursor and fragment mass tolerance were set at 20 ppm and 50 ppm, respectively. Oxidation of methionine was set as a possible modification, carbamidomethylation of cysteine as a fixed. For X! Tandem we also selected parameters that allowed a quick check for protein N-terminal residue acetylation, peptide N-terminal glutamine ammonia loss or peptide N-terminal glutamic acid water loss. Resulting files were submitted to the Scaffold 4 software (version 4.2.1, Proteome Software, Inc, USA) for validation and further analysis. We used the local false discovery rate scoring algorithm with standard experiment-wide protein grouping. A 1% FDR threshold was applied to search results from individual datasets. Frequently observed contaminants, such as trypsin, bovine proteins and human keratins, were removed from the results, along with proteins supported by a single unique peptide.

The mass spectrometry proteomics data have been deposited to the ProteomeXchange Consortium (http://proteomecentral.proteomexchange.org) via the PRIDE partner repository with the dataset identifier PXD013509.

For identification of the mass spectrometry hits, genomes of *M. tuberculosis* H37Rv (NC_000962.3) and RUS_B0 (CP030093.1) strains were annotated. A six-frame translated database of RUS_B0 strain containing translated sequences from stop to stop 6 codons was created by using Artemis v 16.0.0 program^45^. We used ATG, GTG and TTG as possible start codons, and a minimum length of ORF was set to 100 amino acids. Obtained database included 74,488 protein-coding sequences. Twenty-six contaminants (e.g. keratins, BSA, trypsin) were added to the all databases that were used for the MS/MS ion search. Identification after information-dependent acquisition was performed with Sciex ProteinPilot software (version 4.5, AB Sciex, Canada) against a database of H37Rv with addition of newly discovered sequences. A cut-off of 1% local FDR at protein level was applied.

Spectral count per protein data was exported from Scaffold. The data was normalized by dividing each value by average spectral-count for the corresponding biosample. Differences in the abundance of a protein between the three biological replicates of *M. tuberculosis* H37Rv and all replicate of RUS_B0 were evaluated by dividing for each protein average abundance in RUS_B0 strain by average abundance on H37Rv strain. If the difference was more than 2 times, then the protein was considered to be differentially expressed.

#### Proteogenomic analysis

Peptides mapping to multiple places in the RUS_B0 genome were not used for proteogenomic analysis. GSSPs were identified by excluding peptides which mapped to known proteins from the six-frame translated database of RUS_B0. GSSPs present in two or more cultures were considered as reliably identified. Further ORFs, which contained detected GSSPs, have been checked for the presence of protein-coding *M. tuberculosis* RUS_B0 annotated sequences. ORF was considered as identified if i) at least two GSSPs existed; ii) it contained one GSSPs and one annotated protein-coding sequence; iii) it contained one GSSPs and the intersection on coordinates with annotated pseudogene within the chain. GSSPs were checked for the exact entry in the NCBInr (http://www.ncbi.nlm.nih.gov/protein/). This allowed checking the absence of peptides from the samples, previously measured on a mass spectrometer. Corrected and novel genes obtained using GSSPs were checked for their conservation across *M. tuberculosis* complex using protein blast tool. In order to find all possible transcribed ORFs, we assembled RUS_B0 transcriptome *de novo* using rnaSPAdes v3.11^46^. Then all possible ORFs were found in the assembled transcripts using stand-alone version of NCBI’s ORFfinder tool (https://www.ncbi.nlm.nih.gov/orffinder/) (output ORFs on both strands, genetic code Table 11 - Archaea and Bacteria, start codon to use: ATG and alternative initiation codons) and their similarity to the custom database of proteins containing GSSPs were checked by using blast v2.6.0 package. Blast results were filtered using the following criteria: percentage of identical matches −99%, e-value < 0.01.

## Supplementary information

Supporting Information Figures

Table S1

Table S2

Table S3

Table S4

Table S5

Table S6

Table S7

Table S8

Table S9
