# Author Correction: System OMICs analysis of *Mycobacterium tuberculosis* Beijing B0/W148 cluster

**DOI:** 10.1038/s41598-020-61261-2

**Published:** 2020-03-06

**Authors:** Julia Bespyatykh, Egor Shitikov, Andrei Guliaev, Alexander Smolyakov, Ksenia Klimina, Vladimir Veselovsky, Maya Malakhova, Georgij Arapidi, Marine Dogonadze, Olga Manicheva, Dmitry Bespiatykh, Igor Mokrousov, Viacheslav Zhuravlev, Elena Ilina, Vadim Govorun

**Affiliations:** 1 Federal Research and Clinical Centre of Physical-Chemical Medicine, Moscow, Russian Federation; 2 Shemyakin-Ovchinnikov Institute of Bioorganic Chemistry of the Russian Academy of Sciences, Moscow, Russian Federation; 3 Moscow Institute of Physics and Technology (State University), Dolgoprudny, Russian Federation; 4 Research Institute of Phtisiopulmonology, St. Petersburg, Russian Federation; 5 St. Petersburg Pasteur Institute, St. Petersburg, Russian Federation

Correction to: *Scientific Reports* 10.1038/s41598-019-55896-z, published online 17 December 2019

The original version of this Article contained typographical errors throughout the main body of the text.

Within the Abstract,

“Completed genome sequence of RUS_B0 (CP020093.1) and a collection of WGS for 394 cluster strains were used to describe the main genetic features of the population”

now reads:

“Completed genome sequence of RUS_B0 (CP030093.1) and a collection of WGS for 394 cluster strains were used to describe the main genetic features of the population”

Within the Results section under subheading ‘Transcriptomic analysis’,

“Transcriptional regulator WhiB6 is a part of PhoP regulon and has a cluster-specific substitution A253S.”

now reads:

“Transcriptional regulator WhiB6 is a part of PhoP regulon and has a cluster-specific substitution T51P.”

Additionally, within the Discussion,

“This gene contains a cluster-specific polymorphic site, leading to the amino acid substitution A253S in the appropriate protein.”

now reads:

“This gene contains a cluster-specific polymorphic site, leading to the amino acid substitution T51P in the appropriate protein.”

Finally, the Figure legends of Figures 3 and 4, respectively, were inadvertently switched.

The legend for Figure 3:

“Multi-omics analysis of *ethA* gene and its product in H37Rv and RUS_B0 strains. The horizontal axis represents a schematic *ethA* gene (cyan) and protein (yellow), vertical axis represents transcript coverage (blue) and peptides (green). The red square indicates a frame shift mutation in the RUS-B0 strain genome.”

now reads:

“Representative functional clusters for differential genes based on transcriptomic data. Blue color indicates down-regulated genes. Red color indicates up-regulated genes. (**A**) Distribution of genes with diff expression in the main categories; (**B**) Detail representation of genes with diff expression in «biological process» category.”

The legend for Figure 4:

“Representative functional clusters for differential genes based on transcriptomic data. Blue color indicates down-regulated genes. Red color indicates up-regulated genes. (A) Distribution of genes with diff expression in the main categories; (B) Detail representation of genes with diff expression in «biological process» category.”

now reads:

“Multi-omics analysis of *ethA* gene and its product in H37Rv and RUS_B0 strains. The horizontal axis represents a schematic *ethA* gene (cyan) and protein (yellow), vertical axis represents transcript coverage (blue) and peptides (green). The red square indicates a frame shift mutation in the RUS-B0 strain genome.”

These errors have now been corrected in the HTML and PDF versions of this Article.

