# Supplementary material for: System OMICs analysis of Mycobacterium tuberculosis Beijing B0/W148 cluster
Source: Sci Rep. 2019 Dec 17;9:19255. doi: 10.1038/s41598-019-55896-z (PMC6917788; doi:10.1038/s41598-019-55896-z)
Supplement: Supplementary file 1 — Supporting Information Figures [file 41598_2019_55896_MOESM1_ESM.pdf]

System OMICs analysis of *Mycobacterium tuberculosis* Beijing B0/W148 cluster

Julia Bespyatykh[1*], Egor Shitikov[1], Andrei Guliaev[1], Alexander Smolyakov[1,3], Ksenia Klimina[1], Vladimir Veselovsky[1], Maya Malakhova[1], Georgij Arapidi[1,2,3], Marine Dogonadze[4], Olga Manicheva[4], Dmitry Bespiatykh[1], Igor Mokrousov[5], Viacheslav Zhuravlev[4], Elena Ilina[1], Vadim Govorun[1]

1. Federal Research and Clinical Centre of Physical-Chemical Medicine, Russian Federation
2. Shemyakin-Ovchinnikov Institute of Bioorganic Chemistry of the Russian Academy of Sciences, Moscow, Russian Federation
3. Moscow Institute of Physics and Technology (State University), Dolgoprudny, Russian Federation
4. Research Institute of Phtisiopulmonology, St. Petersburg, Russian Federation
5. St. Petersburg Pasteur Institute, St. Petersburg, Russian Federation

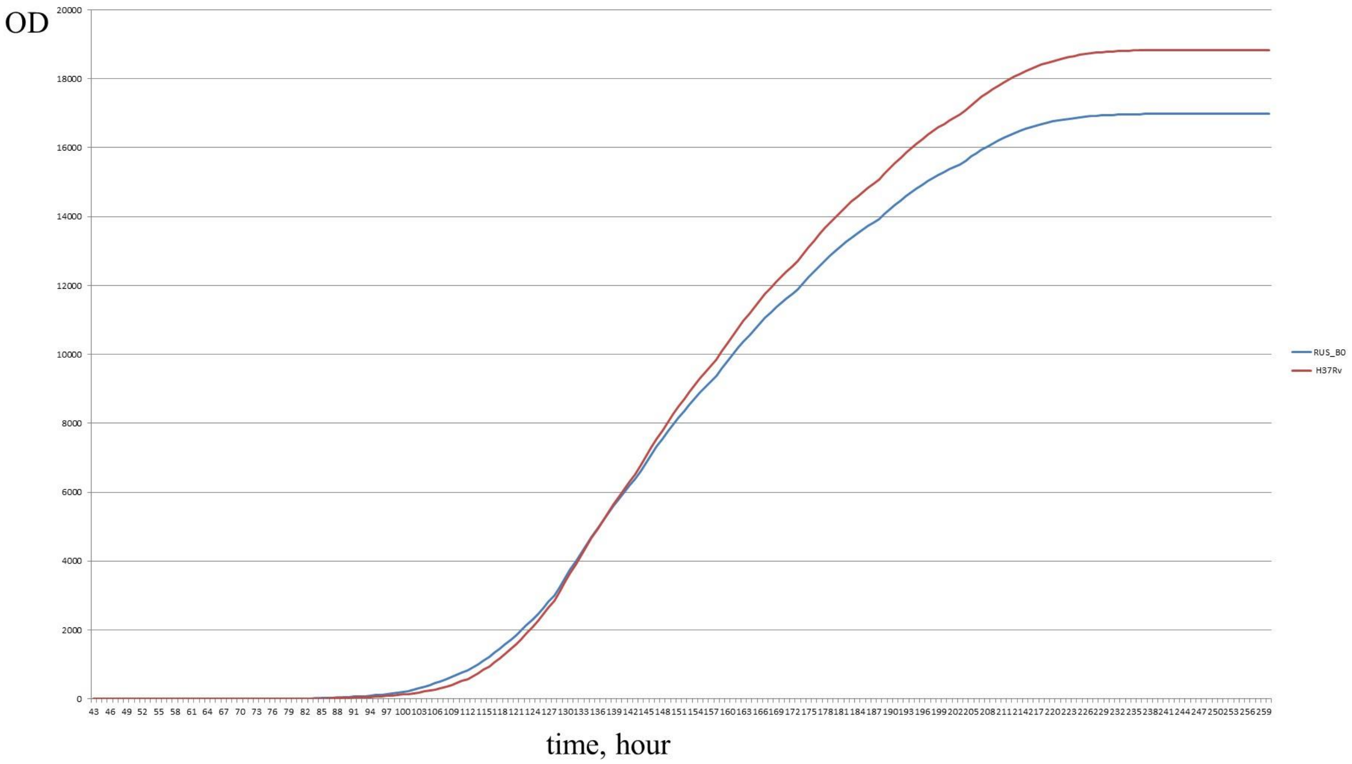
OD
20000
18000
16000
14000
12000
10000
8000
6000
4000
2000
0
time, hour
RUS_B0
H37Rv

Figure S1. Growth curve of *M. tuberculosis* RUS_B0 and H37Rv

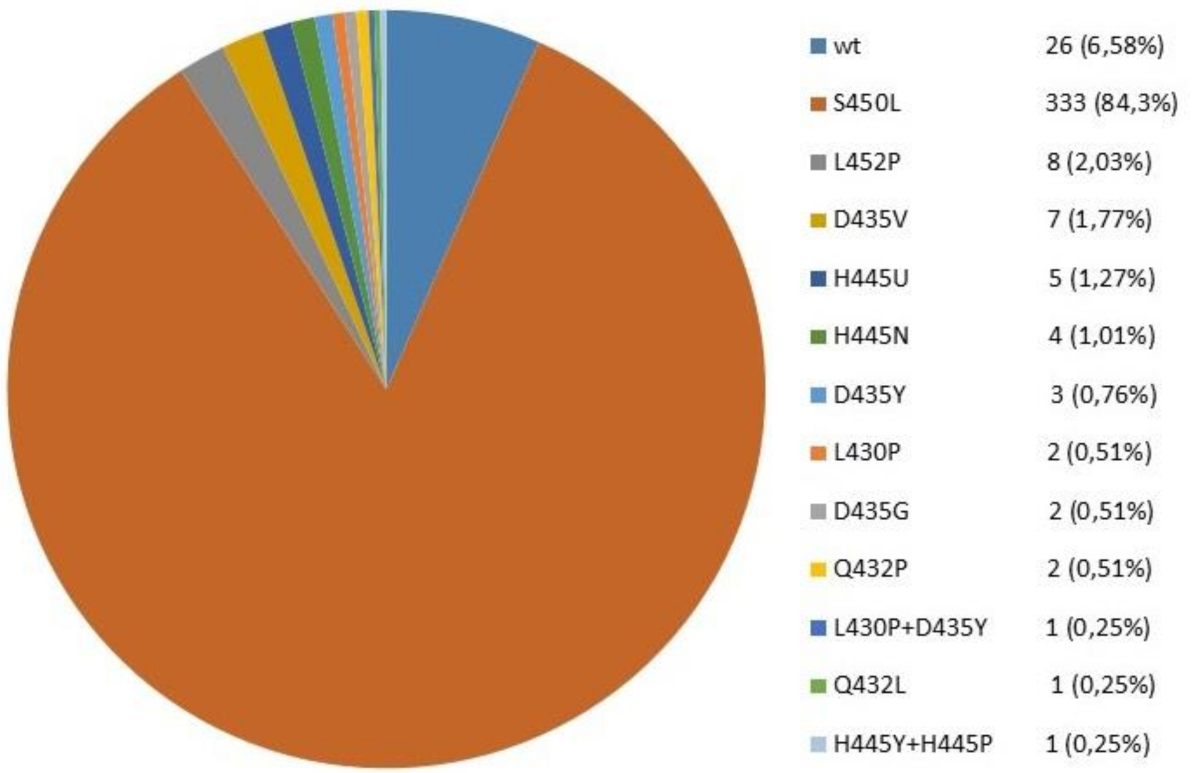
wt 26 (6,58%)
S450L 333 (84,3%)
L452P 8 (2,03%)
D435V 7 (1,77%)
H445U 5 (1,27%)
H445N 4 (1,01%)
D435Y 3 (0,76%)
L430P 2 (0,51%)
D435G 2 (0,51%)
Q432P 2 (0,51%)
L430P+D435Y 1 (0,25%)
Q432L 1 (0,25%)
H445Y+H445P 1 (0,25%)

Figure S2. Prevalence of rifampicin resistance-associated RpoB substitutions in Beijing B0/W148 strains
